# Dictionary of Parasitology

**DOI:** 10.3201/eid1202.051244

**Published:** 2006-02

**Authors:** Mark Eberhard

**Affiliations:** * Centers for Disease Control and Prevention, Atlanta, Georgia, USA

**Keywords:** parasitology, virology, book review

The authors' intent in writing this dictionary is to provide a concise, clear, up-to-date, accurate use of terms to be used when communicating scientific information in the field of parasitology. This exhaustive text, with more than 11,500 entries, is at first read simply an alphabetized collection of names of organisms and terms associated with the science of parasitology. Upon closer reading, however, one spends more and more time going page by page either refreshing forgotten terminology, or learning new meaning for a particular term or disease. For the student of words, both newcomers to the field or seasoned hands, this book will provide useful information. Some concerns exist, such as continued use of outdated names, e.g., *Dipetalonema* for a number of filarial infections that have been correctly placed in the genus *Mansonella* for >20 years. There are also some gaps, such as the absence of an important genus of microsporidia, *Enterocytozoon*, but overall, readers will be able to find definitions for common and eclectic terms. The dictionary covers a wider range of terms than parasitology; some virology terms, such as Aino virus, are included, as are some far afield terms, including hundredweight, hydrogen half-cell, and zwitterions. These additions add to the level of interest as the reader leafs from page to page looking for familiar friends and making new acquaintances. Zwitterions, in case you have forgotten, are ions that carry both a positive and negative charge.

